# Estimating genomic diversity and population differentiation – an empirical comparison of microsatellite and SNP variation in *Arabidopsis halleri*

**DOI:** 10.1186/s12864-016-3459-7

**Published:** 2017-01-11

**Authors:** Martin C. Fischer, Christian Rellstab, Marianne Leuzinger, Marie Roumet, Felix Gugerli, Kentaro K. Shimizu, Rolf Holderegger, Alex Widmer

**Affiliations:** 1 ETH Zürich, Institute of Integrative Biology, Universitätstrasse 16, 8092 Zürich, Switzerland; 2 WSL Swiss Federal Research Institute, Zürcherstrasse 111, 8903 Birmensdorf, Switzerland; 3 Institute of Evolutionary Biology and Environmental Studies and Institute of Plant Biology, University of Zurich, Winterthurerstrasse 190, 8057 Zürich, Switzerland

**Keywords:** Microsatellites, SSR, *Arabidopsis halleri*, Genetic diversity, Expected heterozygosity, SNPs, Population genomics, Whole-genome re-sequencing, Pool-Seq, Conservation units

## Abstract

**Background:**

Microsatellite markers are widely used for estimating genetic diversity within and differentiation among populations. However, it has rarely been tested whether such estimates are useful proxies for genome-wide patterns of variation and differentiation. Here, we compared microsatellite variation with genome-wide single nucleotide polymorphisms (SNPs) to assess and quantify potential marker-specific biases and derive recommendations for future studies. Overall, we genotyped 180 *Arabidopsis halleri* individuals from nine populations using 20 microsatellite markers. Twelve of these markers were originally developed for *Arabidopsis thaliana* (cross-species markers) and eight for *A. halleri* (species-specific markers). We further characterized 2 million SNPs across the genome with a pooled whole-genome re-sequencing approach (Pool-Seq).

**Results:**

Our analyses revealed that estimates of genetic diversity and differentiation derived from cross-species and species-specific microsatellites differed substantially and that expected microsatellite heterozygosity (SSR-*H*_e_) was not significantly correlated with genome-wide SNP diversity estimates (SNP-*H*_e_ and *θ*_Watterson_) in *A. halleri*. Instead, microsatellite allelic richness (*A*_r_) was a better proxy for genome-wide SNP diversity. Estimates of genetic differentiation among populations (*F*_ST_) based on both marker types were correlated, but microsatellite-based estimates were significantly larger than those from SNPs. Possible causes include the limited number of microsatellite markers used, marker ascertainment bias, as well as the high variance in microsatellite-derived estimates. In contrast, genome-wide SNP data provided unbiased estimates of genetic diversity independent of whether genome- or only exome-wide SNPs were used. Further, we inferred that a few thousand random SNPs are sufficient to reliably estimate genome-wide diversity and to distinguish among populations differing in genetic variation.

**Conclusions:**

We recommend that future analyses of genetic diversity within and differentiation among populations use randomly selected high-throughput sequencing-based SNP data to draw conclusions on genome-wide diversity patterns. In species comparable to *A. halleri*, a few thousand SNPs are sufficient to achieve this goal.

**Electronic supplementary material:**

The online version of this article (doi:10.1186/s12864-016-3459-7) contains supplementary material, which is available to authorized users.

## Background

Genetic diversity is essential for organisms to adapt to changing environmental conditions and is recognised as a key component of biodiversity (e.g. [1, 2]). Microsatellite markers (also known as simple sequence repeats, SSRs) are a widely used marker system to estimate genetic diversity in population genetic studies and are often implicitly assumed to reflect the genome-wide diversity of a taxon [3]. The use of microsatellites has increased linearly since their detection in the 1980s [4], and they are nowadays extensively applied, for example in conservation genetics (e.g. [5]), forensic DNA profiling, paternity analyses, and studies of neutral genetic population structure (for reviews see [3, 6, 7]). However, the challenge of correctly interpreting microsatellite data is often strongly underrated [8], and the question whether a limited number of microsatellite markers accurately reflects genome-wide diversity remains a contentious issue (e.g. [9–12]). Single nucleotide polymorphisms (SNPs) on the basis of traditional DNA sequencing [13] have long been known, but in contrast to microsatellites, were relatively rarely used in population genetics until recently because of the difficulties associated with their characterization and genotyping in non-model organisms [14]. Moreover, their (mostly) bi-allelic state limits the information content per locus compared to the more polymorphic microsatellite markers [15–17]. In recent years, the use of SNPs has been exponentially increasing [7], mainly because newly developed high-throughput sequencing techniques can efficiently be applied to a wide range of organisms. These techniques allow for the identification of thousands to millions of unbiased SNPs, and the simultaneous estimation of SNP frequencies across the genomes of individuals, populations and species [18–20].

Microsatellites have unique properties that distinguish them from the rest of the genome, and these should be taken into consideration when analysing and interpreting them [8]. Microsatellites are codominant markers and typically consist of simple sequence repeats varying in length between one and six base pairs. Their variability originates from DNA polymerase slippage during replication, leading to the formation of shorter or longer alleles (for further details see [21–23]). In plants, microsatellite mutation rates range between 10^−6^ and 10^−2^ per locus and generation (for a review see [24]), thus varying approximately 10,000-fold, and are affected by various factors, including repeat type, repeat copy number, marker location in the genome, and taxon [23]. In contrast, spontaneous mutation rates for SNPs only vary about 100-fold [25]. Knowledge of direct estimates of SNP mutation rates is limited, but the rate has been accurately estimated e.g. in *Arabidopsis thaliana* to be 7 × 10^−9^ substitutions per site per generation [26]. Microsatellite mutation rates are therefore several orders of magnitude higher and much more variable than those of SNPs. In combination with the often small number of markers used, microsatellite-based studies typically sample a narrow fraction of the genome with unusually high mutation rate [21]. This may be aggravated when only the most polymorphic microsatellite markers are selected for further analysis after initial screening of a small subsample of individuals or populations. Estimates of genetic diversity may then suffer from ascertainment bias [15, 27]. Additionally, amplification variation of primers [28] and fragment size homoplasy [29] potentially reduce the accuracy of genetic estimates inferred from microsatellite markers. The use of microsatellite markers may thus lead to estimates of genetic diversity and differentiation that do not well reflect genome-wide patterns of variation.

Despite these potential caveats, a large number of studies has relied on microsatellite markers to estimate genetic diversity and genetic differentiation, not only within and among populations, but also among species (e.g. [3, 7, 10, 30]). In a conservation context, microsatellites are also used to identify conservation units (CUs), whose genetic variation and distinctness is potentially relevant for species survival (e.g. [5, 31, 32]). Well-known case studies are the Florida panther [33, 34] or the African elephant from Eritrea [35], for which management decisions were taken based on genetic data derived from few microsatellites.

To date, only few studies have explored in detail to what degree microsatellite variation reflects genetic variation at other nuclear loci, and which genetic diversity estimator for microsatellites provides the most accurate prediction of genome-wide diversity. Positive but sometimes weak correlations between expected microsatellite heterozygosity (SSR-*H*_e_) and SNP diversity in nuclear gene sequences have been reported at the population level in salmon [11, 36, 37] and several carnivore species [10], as well as different rice varieties and sheep breeds [38, 39]. Most of these studies, however, have investigated only a limited number of SNPs (ranging from tens to a few thousand). The outcome of the comparison of SNP versus microsatellite diversity in these studies was strongly affected by the number of SNP markers used. Studies in which low SNP numbers (<300) were compared to microsatellites found that the latter had more power to infer differences in genetic summary statistics [10, 38–46] or found similar results when approximately 400 SNPs were used [47]. In contrast, studies using larger numbers of SNPs (~3000) found that SNPs performed better than microsatellites [11, 12, 37]. Many of these studies used existing genotyping arrays for SNP detection. These may, however, cause ascertainment bias as a consequence of the overrepresentation of common SNPs [8]. To date, no unbiased whole-genome re-sequencing approach has been used for comparison. Studies based on reduced representation libraries (e.g. restriction-site associated DNA sequencing; RADseq), which sample a subset of all SNPs of the genome, showed that SNPs have more power than microsatellites, e.g. to detect heterozygosity–fitness correlations in natural populations of oldfield mice [9]. Further, demographic inferences drawn from RADseq-derived SNPs in bumble bees reflected important long-term differences in population size better than microsatellites, which instead signalled either recent demographic changes or mutational processes [48].

Because of the widespread application of microsatellite markers both in basic research and practical conservation, it is important to evaluate the tenet that microsatellite variation adequately reflects genome-wide genetic diversity, especially for situations in which only a limited number of markers are used, as is often the case in conservation genetics, where on average only 12 microsatellites are used per study [49]. It is further relevant to evaluate the power of next-generation sequencing (NGS) based genotyping approaches to infer genome-wide diversity and population structure, e.g. to estimate the number of SNPs required to achieve accurate and consistent estimates of genome-wide diversity.

We used two types of microsatellite markers (markers developed for the same species and cross-species markers) as well as genome-wide SNP variation in the meadow rock cress, *Arabidopsis halleri* (L.), to compare estimates of genetic diversity and differentiation. Overall, we genotyped 180 individuals of *A. halleri* from nine natural populations using 20 microsatellite markers, which is above the average number of microsatellites typically used in population and conservation genetic studies [49]. We compared them to a pooled whole-genome re-sequencing approach (Pool-Seq; [50, 51]) and tested whether estimates of genetic variation derived from microsatellite polymorphisms are valid and useful proxies of genome-wide genetic variation and differentiation. Specifically, we tested whether estimates from both marker types were correlated (relative comparison) and had similar absolute values (absolute comparison). Further, we used down-sampling to assess how many random and presumably unlinked SNPs are required to calculate accurate estimates of genome-wide diversity.

## Methods

### Study system

*Arabidopsis halleri* is a perennial, insect-pollinated, strictly outcrossing and functionally self-incompatible herb [52] with a wide geographic distribution from central Europe to eastern Asia [53]. It grows in diverse habitats including mountain slopes, grassy meadows, forest margins and rocky crevices [54, 55] and has been widely used as a model to study heavy metal tolerance (e.g. [55, 56]). The species is diploid with 2n = 16 [54] and has an estimated genome size of 255 Mbp [57].

### Sampling and DNA extraction

Leaf tissue from 20 individuals each of nine populations of *A. halleri* was sampled in south-eastern Switzerland and northern Italy (Additional file 1: Table S1). The selected samples size per population should allow to accurately estimate population-specific genetic diversity and differentiation [58]. A minimal distance of two, but preferably 4 m, was maintained between collected individuals, as genetic structure and diversity may be affected by clonal growth when plants are separated by less than one meter [59]. Leaf samples were dried in silica gel, and DNA was extracted with the DNeasy Plant Mini Kit (Qiagen, Hilden, Germany) according to the manufacturer’s protocol. DNA concentrations were measured with a Qubit® 1.0 Fluorometer (dsDNA BR, Carlsbad, USA), and DNA quality was examined using a NanoDrop 8000 Spectrophotometer (Thermo Scientific, Waltham, USA) as well as 1.5% agarose gels stained with GelRed (Biotium, Hayward, USA).

### Microsatellite analyses

The 180 samples were genotyped using 20 microsatellite markers in three multiplex PCRs, each amplifying either six or eight microsatellite markers (Additional file 2: Table S2). The first two multiplex sets included 12 microsatellite loci that were originally developed for *A. thaliana* [52, 60, 61], hereafter referred to as “cross-species” microsatellites. Some of these primer sequences were adapted to *A. halleri* by comparing them to our own *de-novo* assembly of the *A. halleri* genome [51] using IGV 2.1 [62], identifying potential mismatches and changing the primer sequences accordingly (Additional file 2: Table S2). Further, eight microsatellite primer pairs that were specifically developed for *A. halleri* [63] were combined in a third multiplex set. These markers are hereafter referred to as “species-specific” microsatellites. Detailed lab protocols can be found in the Additional Methods (Additional file 3). Alleles were called using GeneMapper 4.1 (Applied Biosystems).

### Estimating microsatellite-based genetic diversity

For every marker and population, we assessed the following population genetic parameters. The inbreeding coefficient *F*_IS_ and its *p*-value, which indicate whether markers or populations deviate from Hardy–Weinberg equilibrium, were calculated with GenoDive 2.0b23 [64] using the heterozygosity-based *G*_IS_ statistic with 999 permutations and applying Bonferroni correction for multiple testing. Null allele frequencies were calculated with FreeNA [28]. Pairwise values of genetic differentiation among populations, *F*_ST_, were calculated based on allele identity with Genepop 4.2.2 [65, 66], whereas allele frequencies, expected heterozygosity (SSR-*H*_e_) and mean number of alleles (allelic richness, *A*_r_) per locus were quantified with Genetix 4.05 [67]. We consistently genotyped 20 individuals per population, therefore, *A*_r_ did not have to be corrected with a rarefaction approach. All population parameters were computed for three different marker sets including (i) all, (ii) the cross-species, and (iii) the species-specific microsatellite markers (Additional file 2: Table S2). To infer marker bias, we tested for quantitative differences in estimates of SSR-*H*_e_ and *A*_r_ estimated in each population from cross-species and species-specific markers using a paired *t*-test (function ‘t.test’) in R 3.2 [68]. For the relative comparison of SSR-*H*_e_ derived from cross-species and species-specific markers, we used a Pearson’s correlation test (function ‘cor.test’) in R. To test whether population-specific estimates of SSR-*H*_e_ obtained from the different microsatellite types differ [69], we used a pairwise Wilcoxon signed-rank test (function ‘pairwise.wilcox.test’) in R. By plotting SSR-*H*_e_ medians and quantiles for each population, we inferred whether non-significant differences were caused by high variance. When the absence of significant differences between populations was obviously caused by overly high variances in the genetic diversity estimates computed per microsatellite marker and population, we interpreted this as variance bias. *P*-values were adjusted for multiple testing using Bonferroni correction.

### Pool-Seq and Illumina read processing

Pooled next-generation sequencing (Pool-Seq) has been shown to produce accurate population-specific allele frequencies [20, 51, 70]. For NGS, individually extracted high-quality DNA was equimolarly pooled using the same 20 individuals from the nine populations as for the microsatellite genotyping presented above. These nine pools were high-throughput sequenced and further processed as described below and, in more detail, in Fischer et al. [50]. For a subset of SNPs and populations of the present dataset, the accuracy of exactly the same Pool-Seq approach had been validated [51]: differences in estimates of population-specific allele frequencies compared to those from individual genotyping were on average less than 4%. Library preparation (~170–250 bp insertion size; 100-bp paired-end reads) and sequencing on an Illumina HiSeq2000 (Illumina, San Diego, USA) were performed by GATC Biotech (Constance, Germany) and the Quantitative Genomics Facility (D-BSSE, ETH Zürich, Switzerland). Forward and reverse raw reads were trimmed for tags and adaptors with Cutadapt [71]. Phred-type quality scores Q20 were used for quality trimming with the FASTX toolkit (http://hannonlab.cshl.edu/fastx_toolkit). The separately trimmed forward and reverse reads were then re-synchronized to pairs with an in-house perl script. Only paired sequences were used for further analysis [50].

### Read mapping, SNP calling and genome-wide population genetic estimates

To estimate genome-wide genetic diversity and differentiation for all nine populations of *A. halleri*, reads were mapped to the *A. thaliana* reference genome (TAIR10, from which organellar DNA was excluded [72–74]) using BWA aln, allowing for 10% mismatch, and sampe [75]. All ambiguously mapped reads were removed and the remaining high-quality reads were sorted with SAMtools 0.1.18 [76]. SNPs were called for the nine populations by producing mpileup files with SAMtools (for details see [50, 76]).

To obtain population-specific genome-wide estimates of genetic diversity, we first calculated Watterson’s theta (*θ*_Watterson_), an estimator that takes into account the number of segregating sites to estimate the population mutation rate. *θ*_Watterson_ was calculated on a gene-by-gene basis (only exons) for each population using the gene and exon annotation of TAIR10 (GFF3_genes.gff; [74]). The perl script ‘Variance-at-position.pl’ of the software package PoPoolation [77] was used with the mpileup file of each population and the.gtf annotation file (transformed from GFF3_genes.gff file) to calculate exonic *θ*_Watterson_. This approach provides unbiased estimates for pooled samples as it corrects for coverage. Exon based *θ*_Watterson_ is a conservative estimate of genetic diversity as it infers diversity from genomic regions that are predominantly under purifying selection, hence from slightly less diverse regions than the rest of the genome. Exon-based diversity estimates are of direct relevance for the adaptive potential of a population, because exons harbour functionally relevant polymorphisms that allow populations to adapt to changing environments. To accurately estimate allele frequencies for estimating *θ*_Watterson_, minimum counts for minor alleles were set to two to account for sequencing errors, leading to a minor allele frequency threshold of 0.05. The minimum coverage per site within populations was set to 20×, which mimics the number of individuals. To further correct for potential errors caused by repeated sequences, a maximum coverage of 400× per population was used as threshold for SNP identification. In order to be included in the genome-wide estimates of gene diversity, 50% of all SNPs within a gene had to reach the above-mentioned thresholds in all nine populations [50]. For all analyses, pool size per population was set to 40 because 20 diploid genomes were represented in each population pool.

Second, we calculated genome-wide SNP-based expected heterozygosity (SNP-*H*_e_), taking all SNPs into account, not only those located in exons. Mpileup files were synchronized and filtered for base quality (Q20) with the perl script ‘mpileup2sync.pl’ of PoPoolation2 [78]. Next, major and minor allele frequencies were calculated with the script snp-frequency-diff.pl. The coverage threshold was the same as mentioned above, except that the minor allele count was set to four, as all nine populations were jointly used to infer minor allele frequencies [50], leading to a more sensitive, but less error-prone minor allele frequency threshold of 0.011. We only used bi-allelic SNPs and calculated the average genome-wide SNP-*H*_e_ as

$$ \mathrm{S}\mathrm{N}\mathrm{P}{\textstyle \hbox{-} }{H}_e=\frac{1}{n}{\displaystyle \sum_{i=1}^n}2{p}_i\left(1-{p}_i\right) $$

where *n* is the number of SNPs, and *p*_*i*_ is the minor allele frequency of the *i*th allele. This approach assumes Hardy–Weinberg equilibrium within populations.

To infer potential demographic events that could strongly influence genetic diversity within populations, we calculated exome-wide Tajima’s *D* using the TAIR10 gene annotation and the perl script ‘Variance-at-position.pl’ in PoPoolation [77]. It is suggested to use a coverage threshold of less than three times smaller than the pool size [79], which is in our case 13×. A negative genome-wide Tajima’s *D* is indicative of an expansion after a bottleneck, whereas a positive *D* is compatible with a scenario of a decrease in population size [80, 81]. To test whether the average of the resulting distribution of Tajima’s *D* was significantly different from zero, we used *t*-tests against random normal distributions (functions ‘t.test’ and ‘rnorm’ in R) with an average of zero and the same standard deviation as observed in the real data of each population.

Estimates of pairwise population genetic differentiation (*F*_ST_) were calculated with ‘fst-sliding.pl’ in PoPoolation2 [78, 82]. Average values of pairwise *F*_ST_ were calculated using the same parameters as mentioned above for the estimates of SNP-*H*_e_ as explained in detail in Fischer et al. [50].

### Comparisons of genetic diversity and differentiation derived from microsatellites and genome-wide SNPs

To explore associations between estimates of genetic diversity derived from microsatellites and SNPs, we performed Pearson’s correlations (‘cor.test’ in R) of microsatellite-based allelic richness (*A*_r_) and expected heterozygosity (SSR-*H*_*e*_). Estimates of genome-wide SNP diversity were derived from exon sequences (*θ*_Watterson_) and genome-wide SNP expected heterozygosity (SNP-*H*_e_). Further, to account for possible confounding effects due to linkage, associations were also tested for a subset of SNPs, consisting of every 50th SNP.

We performed Mantel tests to check for correlations between values of *F*_ST_ derived from genome-wide SNPs and (i) all, (ii) cross-species, (iii) and species-specific microsatellite markers. The same analysis was used to assess correlations between values of *F*_ST_ derived from species-specific and cross-species microsatellite markers. All analyses were performed with 1001 permutations using Ecodist 1.2.7 [83] in R. Finally, we used paired *t*-tests implemented in R to quantitatively evaluate whether *H*_e_ and *F*_ST_ derived from microsatellite markers and SNPs significantly differ.

### Estimating the number of unbiased SNPs required for accurate estimates of genetic diversity

We used a down-sampling procedure to estimate SNP-*H*_e_ with the aim to infer the required number of randomly selected and unlinked SNP markers to obtain accurate estimates of genetic diversity and to reliably rank populations according to their genetic diversity (e.g. for CU identification). Thus, each population was resampled for the same *k* random SNP markers drawn from the pool of more than 2 million SNPs. For each value of *k* varying between 100 and 400,000, we created 1000 random subsamples of *k* SNP markers (starting from *k* 100 up to 10,000 we sampled *k* at steps of 100 and from *k* 10,000 to 400,000 SNPs we sampled *k* in steps of 1000). We then computed the mean expected heterozygosity (*H*_e_) and 95% confidence intervals for each value of *k* observed in each of the 1000 subsamples. Obtained results were used to draw curves representing the variation of the estimated *H*_e_ as a function of genotyping effort in each population. We then identified the number of SNPs for which the upper and lower confidence intervals for expected heterozygosity (SNP-*H*_e_) fell below ±0.01, ±0.005, and ±0.001.

## Results

### Microsatellite diversity

Twenty microsatellite loci were initially used to characterise 180 *A. halleri* individuals from nine populations. We excluded marker *ah59* from further analyses, because it deviated significantly from Hardy–Weinberg equilibrium and exhibited an estimated null allele frequency of 10% (Additional file 4: Table S3). The remaining 19 microsatellite markers harboured 83 alleles and only 0.3% missing data. Allelic richness per locus (*A*_r_) ranged between 2.2 and 3.1 per population, with an average of 2.71 (±0.29 SD), and expected heterozygosity (SSR-*H*_e_) ranged from 0.025 to 0.717 per microsatellite marker. Further details are given in Table 1 and Additional file 4: Table S3. Population allele frequency distributions were fairly noisy, see Additional file 5: Figure S1A. None of the nine populations showed significant deviation from Hardy–Weinberg equilibrium after Bonferroni correction (Table 1).

**Table 1:** Population genetic parameters inferred from 19 microsatellites and genome-wide SNPs for nine populations of *Arabidopsis halleri*. Allelic richness (*A*_r_)*,* expected heterozygosity (SSR-*H*_e_) and inbreeding coefficient *F*_IS_ including its one-sided *p*-value (i.e. heterozygote deficiency) are given. No *F*_IS_ value was significantly different from zero after Bonferroni correction. *θ*_Watterson_ was calculated for 20,617 genes. Expected heterozygosity (SNP-*H*_e_) was calculated from all SNPs across the genome. Tajima’s *D* was calculated for 22,210 genes, *p*-values refer to deviations from zero (*t*-test)

Population	Microsatellites				SNPs			
	*A*_r_	SSR-*H*_e_	*F*_IS_	*F*_IS_ *p*-values	*θ*_Watterson_	SNP-*H*_e_	Tajima’s *D*	Tajima’s D *p*-values
Aha09	2.7	0.392	0.073	0.051	0.0088	0.154	−0.029	<0.001
Aha11	2.6	0.318	0.043	0.217	0.0081	0.138	−0.114	<0.001
Aha18	2.6	0.360	−0.015	0.386	0.0086	0.152	−0.009	0.743
Aha19	2.8	0.332	−0.084	0.027	0.0086	0.150	−0.033	<0.001
Aha21	2.7	0.404	0.075	0.075	0.0083	0.148	−0.021	0.106
Aha31	3.1	0.387	0.068	0.082	0.0093	0.157	−0.119	<0.001
AhaN1	2.9	0.465	0.081	0.030	0.0092	0.155	−0.169	<0.001
AhaN3	3.1	0.399	0.110	0.010	0.0089	0.154	−0.151	<0.001
AhaN4	2.2	0.343	0.058	0.151	0.0067	0.119	−0.103	<0.001
Mean	2.7	0.378	0.045		0.0085	0.148	−0.083	

**Fig. 1:**
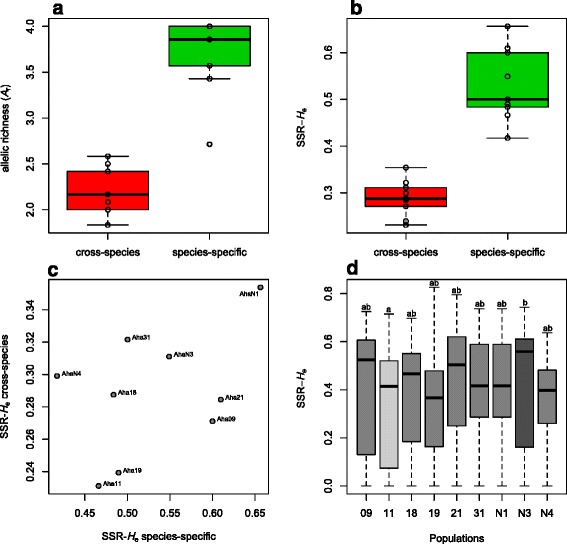
Comparison of estimates of genetic diversity derived from cross-species (developed for *Arabidopsis thaliana*) and species-specific microsatellite markers (developed for *Arabidopsis halleri*) for **a** allelic richness (*A*_r,_ *p* < 0.0001, paired *t*-test) and **b** expected microsatellite heterozygosity (SSR-*H*_e_, *p* < 0.0001, paired *t*-test). **c** Estimates of SSR-*H*_e_ inferred separately from cross-species and species-specific microsatellite markers were not significantly correlated (Pearson’s *r* = 0.439, *p* = 0.237). *Dots* are labelled with population codes (Additional file 1: Table S1). **d** No significantly different estimates of *H*_e_ were observed among populations after Bonferroni correction (pairwise Wilcoxon signed-rank test). Without correction for multiple testing only population Aha11 and AhaN3 showed significantly different estimates of *H*_e_ (indicated with different *colouring* and *letters*)

All twelve cross-species microsatellite markers, initially developed for *A. thaliana*, successfully amplified in *A. halleri. A*_r_ at cross-species microsatellite markers was 2.2 alleles per marker and population and thus significantly lower than *A*_r_ at species-specific markers (3.7 alleles, *p* < 0.0001, paired *t*-test; Fig. 1a). The same pattern (*p* < 0.0001, paired *t*-test; Fig. 1b) was observed for SSR-*H*_e_, which was 0.32 and 0.47, respectively. No significant correlation was observed among estimates of *H*_e_ inferred from cross-species and species-specific microsatellite markers (Pearson’s *r* = 0.439, *p* = 0.237; Fig. 1c).

The variance in the estimates of SSR-*H*_e_ among microsatellite markers within and among population was so high that no significant differences in genetic diversity among populations could be inferred after Bonferroni correction (pairwise Wilcoxon signed-rank test). Without Bonferroni correction, only populations Aha11 and AhaN3 differed significantly in their estimates of *H*_e_ (Fig. 1d). Similar results were found when cross-species and species-specific microsatellites were analysed separately and corrections for multiple testing were performed (Additional file 6: Figure S2).

Average pairwise *F*_ST_ for the 19 microsatellite loci was 0.173 (range: 0.021–0.375); species-specific (mean: 0.169; range: 0.00–0.381) and cross-species microsatellites markers (mean: 0.173; range: 0.021–0.418) did not deviate significantly from each other (*p* = 0.743, paired *t*-test).

### Illumina sequencing and genome-wide diversity

The Illumina sequencing yielded a total of 1,247,939,483 100-bp paired-end reads corresponding to 249,587,896,600 nucleotides. After quality filtering and trimming, 1,197,105,373 paired-end reads were mapped to the *A. thaliana* reference genome (TAIR10), from which organellar DNA was excluded. The average coverage per site, after filtering with the defined thresholds, was 60.7× with a range of population-wise coverage of 52.7 to 69.3 × .

We detected 2,178,204 SNPs, which were used for the calculation of pairwise *F*_ST_, and 2,064,681 bi-allelic SNPs, which were used for the calculation of population-specific SNP-*H*_e_. All populations had even population-specific allele frequency distributions (Additional file 5: Figure S1B). Values of pairwise *F*_ST_ ranged between 0.02 and 0.09, and population-specific SNP-*H*_e_ was between 0.12 and 0.16. Overall, 20,617 and 22,210 genes fulfilled our thresholds of coverage for calculating *θ*_Watterson_ and Tajima’s *D*, respectively. *θ*_Watterson_ ranged from 0.0067 to 0.0093, and Tajima’s *D* values were all slightly negative, ranging from −0.01 to −0.17. Only two of the nine populations showed no significant deviation from zero. In other words, most populations showed weak demographic changes probably related to a bottleneck with later expansion (Table 1 and Additional file 7: Figure S3).

### Comparisons of genetic diversity estimates derived from microsatellites and genome-wide SNPs

**Fig. 2:**
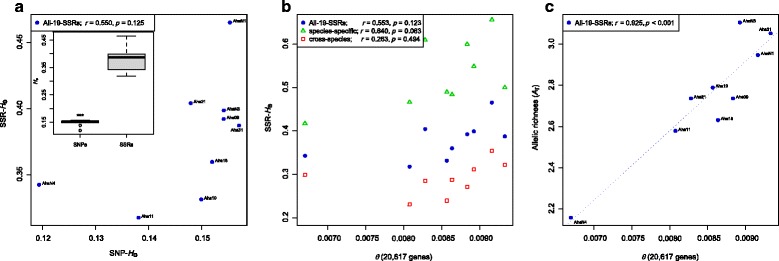
Relationships between population genetic parameters for nine populations of *Arabidopsis halleri* estimated on the basis of 19 microsatellites and 2 million genome-wide single nucleotide polymorphisms (SNPs). **a** Relationship between expected genome-wide SNP heterozygosity (SNP-*H*_e_) and expected microsatellite heterozygosity (SSR-*H*_e_). The *insert* illustrates differences in *H*_e_ between SNPs and microsatellites across the nine *A. halleri* populations. **b** Relationship between genome-wide diversity estimated by average *θ*_Watterson_ across 20,617 genes and expected heterozygosity for all 19 microsatellites (*filled circles*) and separately for species-specific (*open triangles*) and cross-species microsatellites (*open squares*). **c** Comparisons of allelic richness (*A*_r_) with genome-wide SNP variation estimated by *θ*_Watterson_

No significant correlation was observed between population-specific estimates of *H*_e_ derived from microsatellites and genome-wide SNPs (Pearson’s *r* = 0.550, *p* = 0.125; Fig. 2a), independent of whether all SNPs or a subset of presumably unlinked SNPs (every 50th SNP) were used (Pearson’s *r* = 0.572, *p* = 0.108; Additional file 8: Figure S4). Estimates of SNP-*H*_e_ were overall significantly lower than those of SSR-*H*_e_ (paired *t*-test, *p* < 0.0001; Fig. 2a insert). SSR-*H*_e_ was also not significantly correlated with *θ*_Watterson_ (Pearson’s correlation: *r* = 0.553, *p* = 0.123; Fig. 2b). The correlation coefficient was higher when using only species-specific markers (Pearson’s *r* = 0.640, *p* = 0.063), but lower when only cross-species markers were used (Pearson’s *r* = 0.263, *p* = 0.494; Fig. 2b), though neither of the two correlations was significant.

In marked contrast to heterozygosity, *A*_r_ of microsatellite markers was significantly correlated with *θ*_Watterson_ (Pearson’s *r* = 0.925, *p* = 0.0004; Fig. 2c). The correlation based on ranks was still significant (Spearmen’s *ρ* = 0.817, *p* = 0.0108), but slightly weaker than non-ranked comparisons.

**Fig. 3:**
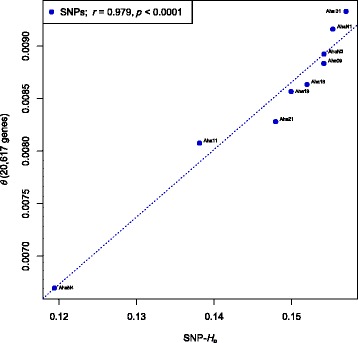
Pearson’s correlation (*dashed line*) between exome-wide *θ*_Watterson_ and genome-wide expected SNP heterozygosity (SNP-*H*_e_) in *Arabidopsis halleri*

The SNP-based genome-wide diversity estimates, *θ*_Watterson_ and SNP-*H*_e_, were highly correlated (Pearson’s *r* = 0.979; *p* < 0.001; Fig. 3), even though values of *θ*_Watterson_ were derived exclusively from coding regions (exons), and estimates of SNP-*H*_e_ were calculated from more than two million SNPs across the entire genome. In fact, *θ*_Watterson_ estimates inferred from introns and intergenic regions were highly correlated to estimates of exon-based *θ*_Watterson_ (Pearson’s *r* = 0.988; *p* < 0.001; see Additional file 9: Figure S5).

### Comparison of genetic differentiation estimates derived from microsatellite versus genome-wide SNP variation

**Fig. 4:**
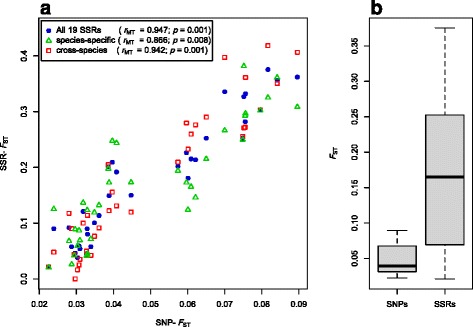
Comparison of pairwise population genetic differentiation (*F*_ST_) among nine populations of *Arabidopsis halleri* estimated with different genetic markers. **a** Comparison of *F*_ST_ derived from 2,178,204 genome-wide SNPs with *F*_ST_ inferred on the basis of all 19 microsatellite markers (*filled circles*), and separately for 12 cross-species (*open triangles*), and seven species-specific microsatellites (*open squares*)*.* Given are correlation coefficients (*r*_MT_) and *p*-values from Mantel tests. **b** Estimates of *F*_ST_ inferred from SNPs were significantly lower than those based on microsatellites (*p* < 0.0001, paired *t*-test)

Mantel tests revealed highly significant correlations between values of pairwise *F*_ST_ derived from genome-wide SNP data and microsatellite markers (Fig. 4). The best correlation was achieved when using all 19 microsatellites (*r*_*MT*_ = 0.947, *p* = 0.001; Fig. 4a). However, values of *F*_ST_ derived from microsatellite markers were 3.35-fold higher than those from SNPs and were significantly different (*p* < 0.0001, paired *t*-test; Fig. 4b). If we split the microsatellite markers into species-specific and cross-species, the correlations were slightly weaker for cross-species microsatellites (*r*_MT_ = 0.942, *p* = 0.001; Fig. 4a) and for species-specific microsatellites (*r*_MT_ = 0.866, *p* = 0.008; Fig. 4a). The correlation among species-specific and cross-species microsatellites was high (*r*_MT_ = 0.829, *p* = 0.004; Additional file 10: Figure S6).

### Estimating the number of unbiased SNPs required for accurate estimates of genetic diversity

**Fig. 5:**
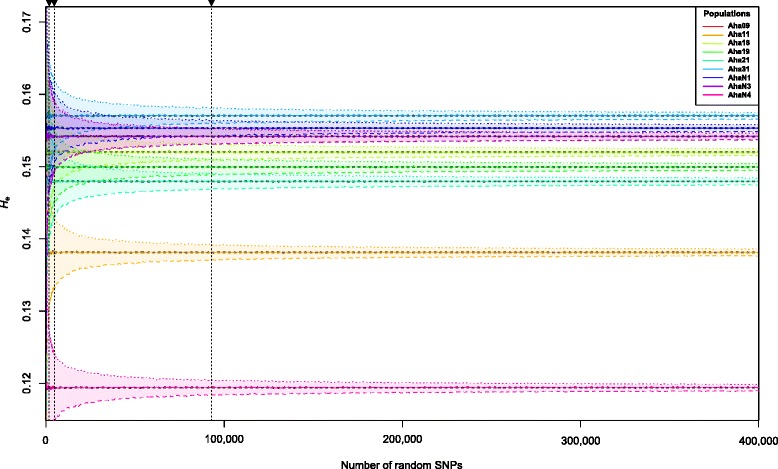
The effect of the number of randomly selected SNP markers on the estimation of *H*_e_ represented for each of nine populations of *Arabidopsis halleri. Light colours* indicate the 95% confidence intervals as inferred from 1000 randomly drawn replicates. *Dashed horizontal lines* (marked by *arrows*) indicate the numbers of SNPs for which 95% confidence intervals for SNP-*H*_e_ are below ±0.01, ±0.005, and ±0.001

SNP down-sampling revealed that the upper and lower confidence intervals for expected heterozygosity (SNP-*H*_e_) fell below ±0.01, ±0.005, and ±0.001 with 1000, 4000 and 93,000 random SNPs, respectively. To accurately and consistently rank all *A. halleri* populations according to their genome-wide diversity (i.e. non-overlapping 95% confidence intervals), 300,000 SNPs were required (Fig. 5). However, populations Aha09 and AhaN3 could not be distinguished, as they have the same genome-wide SNP-*H*_e_ (Table 1).

## Discussion

The application of microsatellite markers is widespread in population and conservation genetic studies. However, NGS-based SNP genotyping approaches are rapidly developing and can be applied to a wide diversity of model and non-model organisms. Our comparative analysis of genetic diversity estimates based on microsatellites and genome-wide SNPs revealed interesting differences. Most importantly, we found no significant correlation between expected microsatellite heterozygosity (SSR-*H*_e_), an estimator of genetic diversity that is widely used and reported in microsatellite studies, and genome-wide SNP diversity (Fig. 2a and b). This finding indicates that SSR-*H*_e_ does not adequately reflect genome-wide genetic diversity in the investigated populations of *A. halleri*. In contrast, microsatellite allelic richness (*A*_r_) was a much better proxy for genome-wide diversity (Fig. 2c). Further, genetic differentiation in terms of *F*_ST_ estimated from microsatellite variation correlated reasonably well with that based on genome-wide SNP data. However, absolute values of the different summary statistics inferred from different marker types varied considerably (Figs. 2a and 4b). Our results do not question the usefulness of microsatellites per se, but point to research questions for which SNPs may be better suited than microsatellite markers, given the availability of robust and cost-effective high-throughput sequencing-based SNP genotyping approaches.

Thanks to the massive advances in sequencing technology, thousands of SNPs can efficiently be genotyped in any given organism, and these may improve our ability to adequately estimate genetic diversity and differentiation. Previous studies found that the potential of SNPs to resolve population genetic structure strongly depends on their number. Indeed, in studies using a low number of assay-based SNPs, microsatellites performed similarly well or better than SNPs [10, 38–47]. However, in studies using larger SNP numbers, especially when they were derived from NGS-based approaches, the relative performance of SNPs clearly improved [9, 11, 12, 37, 48]. These studies show that a large number of SNPs compensates for the lower information content of these typically bi-allelic markers compared to more polymorphic microsatellite markers. Our study shows that a few thousand SNPs are enough to accurately estimate genome-wide diversity in terms of *H*_*e*_ (Fig. 5, see below).

Evidence available from animal species [10, 36, 46] as well as from this study reveals that there is no or only weak congruence between estimates of heterozygosity derived from microsatellites and SNPs. Indeed, theory suggests that an association of heterozygosity estimates between microsatellites and genome-wide SNPs is not expected a priori. According to Ljungqvist et al. [84], this association is shaped by identity disequilibrium, i.e. the non-random association of diploid genotypes between loci. Simulations indicated that a strong positive correlation only emerges when the studied populations are characterized by substantial identity disequilibrium, as was the case in the studies on salmon and carnivores [10, 36]. The absence of this correlation in *A. halleri* suggests that identity disequilibrium is weak or absent, which is compatible with the existence of a strong self-incompatibility system in this species and observed non-significant values of *F*_IS_. This finding indicates that heterozygosity estimates based on microsatellites (SSR-*H*_e_) might not be good surrogates for genome-wide diversity in outbreeding species. Further, we found that the marker-specific variation of SSR-*H*_e_ is too high to distinguish populations based on their levels of genetic diversity (Fig. 1d) and that even among microsatellite markers originally developed for different taxa, important discrepancies can be observed (Fig. 1c and Additional file 6: Figure S2). This variance bias is especially strong when a low number of microsatellites is used, as is the case in many population and conservation genetic studies (on average about 12 markers [49]). The larger sampling variance associated with a limited number of microsatellite markers is evidenced by their allele frequency distributions, which are much noisier than those derived from the SNP data set (Additional file 5: Figure S1).

An alternative estimator to *H*_e_ is microsatellite allelic richness (*A*_r_), one of the simplest estimators of genetic diversity available. *A*_r_ was significantly correlated with genome-wide SNP diversity in our study (Fig. 2c) and thus appears to be a useful proxy of genome-wide genetic diversity. Congruent results of *A*_r_ and SNP diversity were also reported in other studies [10, 36, 49] and might be explained by several reasons. First, SSR-*H*_e_ estimates are based on few markers with noisy allele frequency distributions (Additional file 5: Figure S1A) and represent a proportion, ranging between 0 and 1, whereas allelic richness is an infinite count. Accordingly, one additional allele, especially when it is rare and many alleles are already present, does not strongly influence SSR-*H*_e_, but will affect *A*_r_ estimates, making the latter a more sensitive estimator of diversity. Moreover, especially for microsatellites with a high number of alleles, accurate estimates of population-specific heterozygosity can be problematic [85, 86], and stochasticity may have a strong impact on estimates of *H*_e_ at the lower range of allelic diversity. Finally, *A*_r_ is more sensitive to population bottlenecks than *H*_e_ [49]. Therefore, *A*_r_ better reflects the population’s demographic history and hence is a more relevant estimator of genetic diversity to predict the short-term survival of a population.

Despite the good performance of *A*_r_ as a proxy of genome-wide diversity, it was not sufficient to accurately rank populations according to their genetic diversity (Fig. 2c). Consequently, the identification of conservation units (CUs) or decision taking for conservation actions based on microsatellite-derived rankings of genetic diversity may be misguiding. Nevertheless, using microsatellite-derived *A*_r_ rather than SSR-*H*_e_ provides more accurate estimates of genome-wide genetic diversity derived from a limited number of microsatellite markers. These considerations are strengthened by a simulation study [87], which found allelic richness to be two to four times more powerful than *H*_e_ for the identification of a temporal genetic decline in a population.

A possible reason for the deviation of estimates of genetic diversity derived from microsatellite and genome-wide SNP data could be the influence of very recent demographic changes. As a consequence of their higher mutation rate [21], microsatellites respond more strongly to recent demographic events than genome-wide SNPs [48], and SNPs uncover a different and likely older demographic history. Evidence for this hypothesis has also been presented for bumble bees [48] and may indicate a fruitful application of microsatellites in analyses of populations that may have undergone very recent demographic changes. However, it is important to note that loci with high mutation rate (e.g. microsatellites) may violate demographic model assumptions, such as mutation–migration–drift equilibrium [8]. Further, for the long-term survival of populations, genetic diversity in coding and regulatory sequences is arguably more relevant than microsatellite diversity, because most microsatellites are located in non-coding regions and are mostly selectively neutral, hence of less evolutionary importance. Consequently, estimates of genome-wide SNP diversity better reflect functionally important and potentially adaptive genetic variation [88], and should therefore be used preferentially, especially in conservation genetics studies. In *A. halleri* the long-term demographic history inferred from genome-wide data indicates a bottleneck with later expansion for most populations, as values of Tajima’s *D* were slightly negative and significantly different from zero (Table 1 and Additional file 7: Figure S3).

The origin of the microsatellite markers used, i.e. whether they are species-specific or cross-species markers, may further impact estimates of genetic diversity [27]. In this study, species-specific microsatellites displayed significantly higher *A*_r_ and SSR-*H*_e_ than cross-species markers (Fig. 1a, b) originally developed for *A. thaliana* [52, 60, 61]. Further, species-specific markers resulted in more accurate estimates of genetic diversity (*A*_r_ *;* Fig. 2b), but less accurate estimates of divergence (*F*_ST_; Fig. 4a) among populations of *A. halleri*. Hence, the practicability of microsatellites for population genetic studies is limited and difficult to assess a priori [8].

High estimates of genetic diversity derived from species-specific microsatellite markers may be a consequence of ascertainment bias caused by selecting the most polymorphic markers [15, 27], whereas cross-species microsatellites are mostly chosen based on their amplification success in the study species*.* The consequence of this ascertainment bias is evident in this study, as estimates of SSR-*H*_e_ for cross-species microsatellites are not significantly correlated with SSR-*H*_e_ for species-specific microsatellites (Fig. 1c). These differences further emphasize our inference that marker choice may substantially bias estimates of genetic diversity and may invalidate comparisons between populations or species, most notably when different microsatellite loci are assessed [10].

A different pattern emerges for estimates of population genetic differentiation in terms of pairwise *F*_ST_. We found a significant positive correlation between values of pairwise *F*_ST_ derived from microsatellites and genome-wide SNPs (Fig. 4). Similar findings were reported for salmons and threespine sticklebacks [36, 37, 46]. A reason for the better correlation between estimates of genetic differentiation compared to estimates of genetic diversity may be that more values are involved in pairwise comparisons, and that differences in allele frequencies of the common alleles are more important for the accurate estimation of genetic differentiation than those of rare alleles. Importantly, estimates of *F*_ST_ derived from microsatellites were consistently and substantially higher than those based on genome-wide SNPs. This seems counterintuitive, because multi-allelic microsatellite markers with high mutation rates (and thus high genetic diversity) should cause lower *F*_ST_ values than low-diversity markers like SNPs [89]. However, pooled whole-genome re-sequencing studies with high coverage, such as this one, also detect rare variants; these low-frequency SNPs reduce overall *F*_ST_ [90]. Overall, we consider pairwise population genetic differentiation estimated from microsatellites a useful proxy for genome-wide differentiation, but only in relative and not in absolute terms (Fig. 4b). This finding has serious implications, because absolute values of *F*_ST_ continue to be frequently used to infer indirect estimates of gene flow and migration, even though estimates of gene-flow should not be derived from *F*_ST_ [91]. Further, this marker-specific difference in *F*_ST_ estimates has a major impact on comparative studies of the divergence of quantitative traits, known as *Q*_ST_–*F*_ST_ comparisons, because the inference of the role of natural selection and genetic drift as causes of population genetic differentiation in complex polygenic traits is biased [92].

While our results suggest that microsatellites should not be used for estimating genome-wide heterozygosity, we emphasize that microsatellites remain useful molecular markers for other applications. For example, microsatellites perform very well in genetic stock identification or paternity analysis owing to their high variability [15, 93–96] and may therefore continue to play an important role in molecular ecology. However, before embarking on a molecular analysis, it remains a key issue to carefully assess the inherent strengths and limitations associated with different molecular markers [8]. Only then it is possible to select the most appropriate method for a given ecological or evolutionary question [46].

In contrast to microsatellite-derived data, estimates of genome-wide diversity inferred from whole-genome re-sequencing data, e.g. exome-wide *θ*_Watterson_, intronic and intergenic *θ*_Watterson_, or genome-wide SNP-*H*_e_, were highly correlated with each other and led to the same ranking of populations (Fig. 3; Additional file 9: Figure S5). Even though values of *θ*_Watterson_ were either derived exclusively from coding regions or intronic and intergenic regions, and SNP-*H*_e_ was calculated from positions across the whole genome, their estimates were highly congruent. The slight variation observed among *θ*_Watterson_ and SNP-*H*_e_ (Fig. 3) might be explained by differences in the demographic history among populations (Tajima’s *D* in Table 1), because the demographic history of a population has a stronger influence on *θ*_Watterson_ (the number of segregating sites) than on SNP-*H*_e_ or SNP nucleotide diversity estimates [49], as rare alleles are more likely to be lost during a bottleneck than common ones. Further, we found in our whole-genome re-sequencing study that the confounding effects of genetic linkage are negligible in the highly outcrossing and self-incompatible *A. halleri*, most likely because the small-scale linkage effects are compensated by the large numbers of unlinked SNPs. Thus, our SNP-*H*_e_ estimates based on a subset of putatively unlinked SNPs were nearly identical to the estimates inferred for all SNPs, see Fig. 2a and Additional file 8: Figure S4 . Similar to our genome re-sequencing study, approaches that use reduced representation libraries (e.g. RADseq) to sample a subset of genome-wide SNPs can accurately estimate genome-wide heterozygosity. As a consequence of the much smaller proportion of the genome surveyed with such approaches, however, care should be taken to avoid confounding effects of linkage, for example by considering only one SNP per RAD-locus [97]. For example, the inbreeding coefficient of a known pedigree in oldfield mice showed strong concordance with the inferred estimates of heterozygosity obtained from 13,198 RADseq SNPs [9]. This result indicates that, as long as a sufficiently large number of unbiased NGS-based SNPs is analysed across the genome, SNP estimates accurately reflect genome-wide diversity in natural populations. Therefore, approaches like RADseq [98], Pool-Seq [20] and whole-genome re-sequencing at low coverage [99, 100] are more appropriate than array-based SNP approaches, which may be affected by strong ascertainment bias [17, 101].

An important question to consider in many studies may be the number of SNPs that are needed to estimate genetic diversity. Our down-sampling approach indicated that the number of random SNPs that are required to resolve genetic diversity difference among populations range from 1000 (confidence intervals ± 0.01 SNP-*H*_e_) to 93,000 SNPs (±0.001 SNP-*H*_e_). This number is in the range of SNPs that can be inferred with standard RADseq protocols also in non-model organisms [98]. However, to differentiate among populations with very similar levels of genetic diversity, we required approximately 300,000 SNPs (Fig. 5). Thus, large SNP datasets that are ideally identified *de novo* through NGS approaches (to prevent ascertainment bias) are highly suitable to distinguish, for example, between populations differing in genetic diversity and may therefore support decision-making in conservation management. A further advantage of genome-wide SNP data is that they not only allow one to estimate neutral genetic diversity, but also to identify adaptive genetic variation (e.g. [50, 102–104]), which is considered essential for delimitating conservation units (CUs; [5, 32, 105]). The large technical advances in nucleotide sequencing technology in recent years have not only massively increased the number of nucleotides that can be sequenced per individual or population, but have also led to reduced costs per nucleotide to the extent that screening a handful of microsatellite markers may be as expensive as surveying thousands of SNPs using latest NGS-based genotyping technologies (e.g. [19, 98]).

## Conclusion

This case study in the perennial and outcrossing plant *A. halleri* reveals that genetic diversity estimated from microsatellite markers, notably expected heterozygosity, may not adequately reflect genome-wide genetic diversity estimated from single-nucleotide polymorphisms and may therefore be a poor proxy for genome-wide estimates of genetic diversity. Possible causes include the limited number of microsatellite markers used, marker ascertainment bias, as well as the high variance in microsatellite-derived diversity estimates. Interestingly, microsatellite allelic richness (*A*_r_) was found to be a reasonable proxy for genome-wide diversity, but the absolute ranking of populations was still inconsistent. Estimates of genetic differentiation (*F*_ST_) among populations derived from microsatellites were consistently higher than SNP-based estimates but were significantly correlated with the latter.

Our results do not question the usefulness of microsatellites per se, but point to research questions for which NGS-derived SNPs may be better suited than microsatellite markers, given the availability of robust and cost-effective SNP genotyping approaches based on high-throughput sequencing. As a consequence, we recommend using genome-wide analyses of SNP diversity when the inference and comparison of genetic diversity within and among populations and species is the goal of a study. A few thousand NGS-derived SNPs are sufficient for this purpose and this number of unbiased SNPs can nowadays easily been obtained also for non-model species, for example by using a reduced representation sequencing approach such as RADseq [98].

## Additional files

Additional file 1: Table S1. Sampling locations of the nine study populations of *Arabidopsis halleri*. (PDF 31 kb)
Additional file 2: Table S2. Characteristics of 20 microsatellite markers used for *Arabidopsis halleri*. Source refers to the species for which a microsatellite marker was originally developed. Sequences of forward and reverse primers are shown in 5′ - 3′ direction. The fluorescent dyes used in multiplex PCRs and allele size ranges in base pairs are given for each locus. (PDF 60 kb)
Additional file 3: Additional Methods. (PDF 23 kb)
Additional file 4: Table S3. Locus-specific summary statistics for all 20 microsatellite markers genotyped in *Arabidopsis halleri*. Estimated frequency of null alleles, total number of alleles found among the 180 individuals, expected heterozygosity (SSR-*H*_e_), and inbreeding coefficient *F*_IS_ with one-sided *p*-value (significant heterozygote deficiency indicated in bold) are given. (PDF 28 kb)
Additional file 5: Figure S1. (A) Population specific allele frequency distributions in nine populations of *A. halleri* for 19 microsatellite markers (blue). (B) Minor allele frequency distributions in the same nine populations across 2,064,681 SNPs (green). Histograms are labelled with population codes (Additional file 1: Table S1). (PDF 1421 kb)
Additional file 6: Figure S2. Box-whisker plot of expected heterozygosity calculated for each microsatellite marker (SSR-*H*_e_) genotyped in *Arabidopsis halleri*. For cross-species microsatellites (left, red), no significantly different estimates of *H*_e_ (pairwise Wilcoxon signed-rank test) were observed after Bonferroni correction, as a consequence of the high variance. Without correction for multiple testing, only population Aha11 had a significantly different *H*_e_ estimates from Aha31 and AhaN1 (indicated with different colouring and letters). For species-specific markers (right, green), no significant differences in *H*_e_ estimates were observed after Bonferroni correction. Without multiple corrections, only Aha21 was significantly different from AhaN4. (PDF 225 kb)
Additional file 7: Figure S3. Observed (solid red lines) versus expected (dotted black lines) distributions of Tajima’s *D* for nine populations of *Arabidopsis halleri*. The expected normal distribution consists of 22,210 values with an average of zero and the same standard deviation as the real dataset. *p*-values indicate whether there was a significant deviation of the average from zero using a *t*-test. (PDF 370 kb)
Additional file 8: Figure S4. Relationships between expected SNP heterozygosity (SNP-*H*_e_) and expected microsatellite heterozygosity (SSR-*H*_e_) of *Arabidopsis halleri* estimated on the basis of 19 microsatellites and 41,294 genetically unlinked genome-wide single nucleotide polymorphisms (SNPs). For this analysis only every 50th SNP of the more than 2 million SNPs was used. The median distance among two neighbouring SNPs is 1600 bp on the *A. thaliana* reference genome. (PDF 198 kb)
Additional file 9: Figure S5. Pearson’s correlation (dashed line) between exome-wide *θ*_Watterson_ and *θ*_Watterson_ calculated from introns and intergenic regions in *Arabidopsis halleri*. (PDF 191 kb)
Additional file 10: Figure S6. Comparison of pairwise population genetic differentiation (*F*_ST_) among nine populations of *Arabidopsis halleri* based on 12 cross-species microsatellite markers and seven species-specific microsatellite markers. *r*_MT_ represents the correlation coefficient of the Mantel test and *p* the significance of the correlation. (PDF 181 kb)

## Abbreviations

*A*_r_: Microsatellite allelic richness
*H*_e_: Expected heterozygosity
NGS: Next-generation sequencing
Pool-Seq: Pooled next-generation sequencing
RADseq: Restriction-site associated DNA sequencing
SNP: Single nucleotide polymorphism
SSR: Simple sequence repeat
*θ*_Watterson_: Watterson’s theta
