# Supplementary material for: Estimating genomic diversity and population differentiation – an empirical comparison of microsatellite and SNP variation in Arabidopsis halleri
Source: BMC Genomics. 2017 Jan 11;18:69. doi: 10.1186/s12864-016-3459-7 (PMC5225627; doi:10.1186/s12864-016-3459-7)
Supplement: Additional file 1: Table S1. — Sampling locations of the nine study populations of Arabidopsis halleri. (PDF 31 kb) [file 12864_2016_3459_MOESM1_ESM.pdf]

Additional file 1: Table S1 Sampling locations of the nine study populations of *Arabidopsis halleri*.

Population	Location	Longitude [E]	Latitude [N]	Altitude [m a.s.l.]	Sampling year
Aha09[1]	Vicosoprano (CH)	9°39'31.3''	46°22'9.3''	1403	2010
Aha11[1,2]	Brusio (CH)	10°6'22.0''	46°16'39.0''	1070	2010
Aha18[2]	Chironico (CH)	8°49'33.8''	46°24'57.4''	850	2010
Aha19[1]	Poschiavo (CH)	10°1'21.2''	46°24'40.5''	2308	2010
Aha21[1]	Vicosoprano (CH)	9°37'51.0''	46°22'0.6''	1610	2011
Aha31[1,2]	Castasegna (CH)	9°31'18.2''	46°20'12.5''	790	2011
AhaN1	Brusio (CH)	10°6'2.7''	46°16'31.1''	987	2012
AhaN3	St. Moritz (CH)	9°49'38.4''	46°29'57.3''	2068	2012
AhaN4	Passo di Foscagno (I)	10°13'20.2''	46°29'28.1''	2155	2012

[1] Samples also used in Fischer *et al.* [1]
[2] Samples also used in Rellstab *et al.* [2]

REFERENCES

1. Fischer MC, Rellstab C, Tedder A, Zoller S, Gugerli F, Shimizu KK, Holderegger R, Widmer A. Population genomic footprints of selection and associations with climate in natural populations of *Arabidopsis halleri* from the Alps. Mol Ecol. 2013;22:5594-607.
2. Rellstab C, Zoller S, Tedder A, Gugerli F, Fischer MC. Validation of SNP allele frequencies determined by pooled next-generation sequencing in natural populations of a non-model plant species. PLoS One. 2013;8:e80422.
