# Supplementary material for: Estimating genomic diversity and population differentiation – an empirical comparison of microsatellite and SNP variation in Arabidopsis halleri
Source: BMC Genomics. 2017 Jan 11;18:69. doi: 10.1186/s12864-016-3459-7 (PMC5225627; doi:10.1186/s12864-016-3459-7)
Supplement: Additional file 2: Table S2. — Characteristics of 20 microsatellite markers used for Arabidopsis halleri. Source refers to the species for which a microsatellite marker was originally developed. Sequences of forward and reverse primers are shown in 5′ - 3′ direction. The fluorescent dyes used in multiplex PCRs and allele size ranges in base pairs are given for each locus. (PDF 60 kb) [file 12864_2016_3459_MOESM2_ESM.pdf]

Additional file 2: Table S2 Characteristics of 20 microsatellite markers used for *Arabidopsis halleri*. Source refers to the species for which a microsatellite marker was originally developed. Sequences of forward and reverse primers are shown in 5' - 3' direction. The fluorescent dyes used in multiplex PCRs and allele size ranges in base pairs are given for each locus.

Multiplex set	Locus	Source	Original reference	Forward primer	Reverse primer	Dye[1]	Allele range
A	AthZFPG	*A. thaliana*	[1]	TTGCGTTTCCACATTTGTTT	TGGGTCAATTCACATGTTGAGA*	565	140-153
A	F19K23-483	*A. thaliana*	[1]	GGTCTGATTGCCGTTGTTGC	GATTTCTGTAACATCCCATTTCC*	YY	185-189
A	ICE13	*A. thaliana*	[1]	GATCCTTCACCGGGTCTTG	GTGGTGGAGACTCTTCGAGC	550	226-253
A	MHJ24	*A. thaliana*	[1]	CCGTCCTTGATCCTTGAGATTCTGAG	CAATTCCGAAAATCATATTCATGCACC	550	130-138
A	nga162	*A. thaliana*	[2]	CATGCAATTTGCATCTGAGG	CTCTGTCACTCTTTTCTTCTGG*	YY	78-80
A	nga361	*A. thaliana*	[3]	AGGGTTTTCCCAAAGAGATGA	TCTTGTCCTTTGATTTTAGACCA*	FAM	125-145
B	AthCDPK9	*A. thaliana*	[1]	ATCAATCATTGTCCAAAACTTG*	GAAACTGACTTGAAGAAGGCA*	FAM	87-89
B	ICE10#	*A. thaliana*	[1]	GACTCTTATGGGTAAGCTCCTTG	AACCTCCACAAGTTTCTAAAACAATC*	550	127-141
B	ICE11	*A. thaliana*	[1]	TTTCAAGTTGAGAAGTGGAGTG	AAGATTAGACAGAGTTTAGTGGTC*	565	147-149
B	ICE2#	*A. thaliana*	[1]	GCTACCAGATCCGATGGTAAGATG	CTCGAGTCAAAATTAGGGTTTCG*	YY	136-137
B	ICE5	*A. thaliana*	[1]	CTTGCAACCGCCAACTCAATCG	CTTGTCTCGCTCCCGCACG*	FAM	170-171
B	nga111#	*A. thaliana*	[2]	TGTTTTTTAGGACAAATGGCG	CTGCAGTTGGAAGCTAAAGGG*	YY	105-125
C	ah15	*A. halleri*	[4]	CCAGGAAAGGCAAATCAAGA	AGGGACGCACGATTTTAGTG	NED	146-154
C	ah22	*A. halleri*	[4]	CCAGTTTCGATTTGTTTTACTTTG	AGTTGTGTGATGTAATAAGGTGAAAT	PET	166-177
C	ah27	*A. halleri*	[4]	TTTTTGGCGTTTATCATGTCA	CGATGGTCCACGAATAATTG	FAM	240-246
C	ah44	*A. halleri*	[4]	AGCCCTAGCATCTCCTTTCA	TCGTGTTTGTGCTTGAGTTTG	VIC	83-115
C	ah49	*A. halleri*	[4]	CGGCCACCTCTCTGTAAATC	GAGAACAATGTTGAATTGATTGC	NED	59-71
C	ah59†	*A. halleri*	[4]	GGAAGCAATACTGGGGAAGC	AAAAACCTTAGCCCTTTTGG	VIC	169-191
C	ah79	*A. halleri*	[4]	TGCATTGTGTTGTGTTTCTGC	TCACATGACGCAATATGCAC	PET	82-90
C	ah89	*A. halleri*	[4]	TCCAACACAAGGGGAAAGTC	CCAACCTTGCGAGTTGATTC	PET	235-261

[1] Used fluorescent dyes (565 for ATTO 565, 550 for ATTO 550, and YY for Yakima Yellow)

#Forward and reverse primers were switched compared to the original reference

*Primer sequence was adapted according to own draft genome of *A. halleri* [5] to increase amplification success

† Excluded from final analyses as a consequence of high proportion of null alleles

REFERENCES

1. Clauss MJ, Cobban H, Mitchell-Olds T. Cross-species microsatellite markers for elucidating population genetic structure in *Arabidopsis* and *Arabis* (Brassicaeae). Mol Ecol. 2002;11:591-601.
2. Bell CJ, Ecker JR. Assignment of 30 microsatellite loci to the linkage of *Arabidopsis*. Genomics. 1994;19:137-44.
3. Llaurens V, Castric V, Austerlitz F, Vekemans X. High paternal diversity in the self-incompatible herb *Arabidopsis halleri* despite clonal reproduction and spatially restricted pollen dispersal. Mol Ecol. 2008;17:1577-88.
4. Godé C, Decombeix I, Kostecka A, Wasowicz P, Pauwels M, Courseaux A, Saumitou-Laprade P. Nuclear microsatellite loci for *Arabidopsis halleri* (Brassicaceae), a model species to study plant adaptation to heavy metals. Am J Bot. 2012;99:e49-52.
5. Rellstab C, Zoller S, Tedder A, Gugerli F, Fischer MC. Validation of SNP allele frequencies determined by pooled next-generation sequencing in natural populations of a non-model plant species. PLoS One. 2013;8:e80422.
