# Supplementary material for: Estimating genomic diversity and population differentiation – an empirical comparison of microsatellite and SNP variation in Arabidopsis halleri
Source: BMC Genomics. 2017 Jan 11;18:69. doi: 10.1186/s12864-016-3459-7 (PMC5225627; doi:10.1186/s12864-016-3459-7)
Supplement: Additional file 3: — Additional Methods. (PDF 23 kb) [file 12864_2016_3459_MOESM3_ESM.pdf]

Additional file 3: Additional Methods

Microsatellite lab protocol

Multiplex PCRs for microsatellite markers were conducted with the Type-it® Microsatellite PCR Kit (Qiagen, Hilden, Germany) using labelled forward primers (Microsynth, Balgach, Switzerland; Additional file 2: Table S2). For the cross-species microsatellites, the 11 μL reaction mix contained 3.5 μL RNase-free water, 5.5 μL 2x Multiplex PCR Master Mix, 1.0 μL of primer mix (average of 0.2 μM of each primer in the total reaction volume), and 1.0 μL genomic DNA (2 ng/μL). The cycling protocol consisted of 5 min initial denaturation at 95°C, 28 cycles of 30 s denaturation at 95°C, 90 s primer annealing at 57°C, and 30 s extension at 72°C, and a final extension of 30 min at 60°C. For the species-specific microsatellites, we used a total reaction volume of 10 μL containing 4 μL of 2x Multiplex PCR Master Mix, 2.7 μL of primer mix (average of 0.2 μM of each primer in the total reaction volume), 1.8 of μL RNase-free water, and 1.5 μL of DNA. The cycling protocol was the same as above, except that we used 31 cycles, the annealing temperature was 58°C, and the final extension at 65°C lasted for 45 min. Subsequently, 0.1 μL of PCR product, 0.9 μL RNase-free water, 0.1 μL of GeneScan™-500 LIZ® size standard (Applied Biosystems, Carlsbad, USA), and 9.9 μL of Hi-Di™ formamide (Applied Biosystems) were mixed for fragment analysis on a 3730 DNA Analyzer (Applied Biosystems).
