# Supplementary material for: Estimating genomic diversity and population differentiation – an empirical comparison of microsatellite and SNP variation in Arabidopsis halleri
Source: BMC Genomics. 2017 Jan 11;18:69. doi: 10.1186/s12864-016-3459-7 (PMC5225627; doi:10.1186/s12864-016-3459-7)
Supplement: Additional file 4: Table S3. — Locus-specific summary statistics for all 20 microsatellite markers genotyped in Arabidopsis halleri. Estimated frequency of null alleles, total number of alleles found among the 180 individuals, expected heterozygosity (SSR-H e), and inbreeding coefficient F IS with one-sided p-value (significant heterozygote deficiency indicated in bold) are given. (PDF 28 kb) [file 12864_2016_3459_MOESM4_ESM.pdf]

Additional file 4: Table S3 Locus-specific summary statistics for all 20 microsatellite markers genotyped in *Arabidopsis halleri*. Estimated frequency of null alleles, total number of alleles found among the 180 individuals, expected heterozygosity (SSR-H_e), and inbreeding coefficient F_{IS} with one-sided *p*-value (significant heterozygote deficiency indicated in bold) are given.

Name	Null alleles [%]	No. of alleles	[1]SSR-H_e	F_{IS}	F_{IS} one-sided *p*-value
AthZFPG	2.4	8	0.537	-0.010	0.562
F19K23-483	0.4	4	0.476	-0.077	0.139
ICE13	0.7	6	0.416	-0.029	0.319
MHJ24	3.4	4	0.472	0.023	0.311
nga162	3.4	2	0.358	-0.024	0.434
nga361	5.2	5	0.520	0.145	0.014
AthCDPK9	1.2	2	0.032	0.295	0.135
ICE10	6.1	3	0.401	0.187	0.011
ICE11	0.5	2	0.039	0.136	0.467
ICE2	0.1	2	0.025	-0.118	0.758
ICE5	0.2	2	0.226	-0.080	0.297
nga111	0.2	2	0.059	0.059	0.589
ah15	3.8	5	0.503	0.084	0.059
ah22	2.2	9	0.717	0.001	0.413
ah27	0.8	4	0.515	-0.013	0.459
ah44	3.6	7	0.553	0.096	0.038
ah49	5.3	7	0.503	0.138	0.017
*ah59**	10.0	7	0.508	0.170	**0.001**
ah79	4.5	4	0.485	0.084	0.096
ah89	2.9	5	0.538	0.070	0.137

[1] SSR-H_e for single loci was calculated with GENODIVE [1]
* excluded from further analyses

REFERENCES

1. Meirmans PG, Van Tienderen PH. GenoType and GenoDive: two programs for the analysis of genetic diversity of asexual organisms. Mol Ecol Notes. 2004;4:792-4.
