# Supplementary material for: Estimating genomic diversity and population differentiation – an empirical comparison of microsatellite and SNP variation in Arabidopsis halleri
Source: BMC Genomics. 2017 Jan 11;18:69. doi: 10.1186/s12864-016-3459-7 (PMC5225627; doi:10.1186/s12864-016-3459-7)

Additional file 5: Figure S1 (A) Population specific allele frequency distributions in nine populations of *A. halleri* for 19 microsatellite markers (blue). (B) Minor allele frequency distributions in the same nine populations across 2,064,681 SNPs (green). Histograms are labelled with population codes (Additional file 1: Table S1).

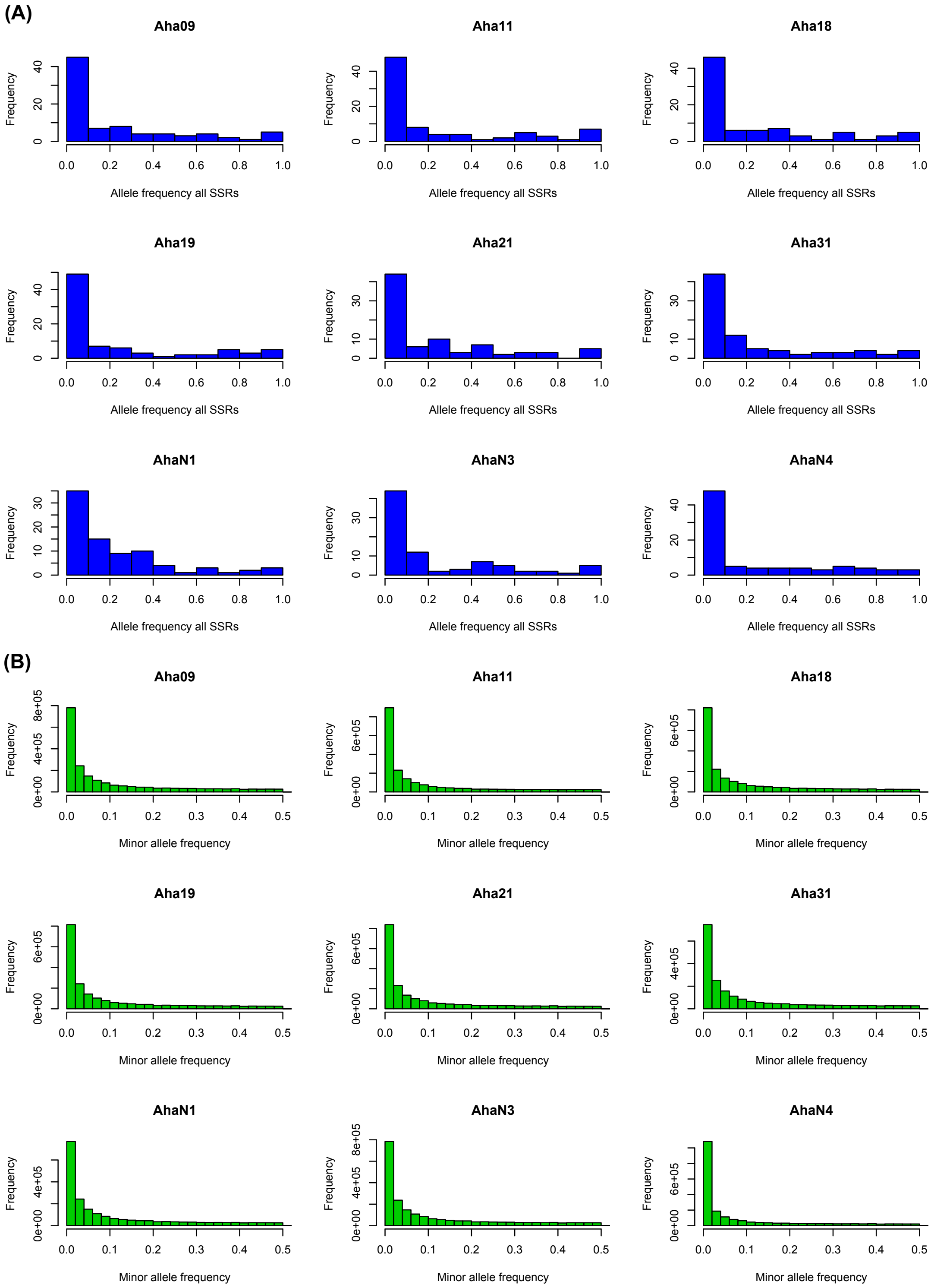

Supplement: Additional file 5: Figure S1. — (A) Population specific allele frequency distributions in nine populations of A. halleri for 19 microsatellite markers (blue). (B) Minor allele frequency distributions in the same nine populations across 2,064,681 SNPs (green). Histograms are labelled with population codes (Additional file 1: Table S1). (PDF 1421 kb) [file 12864_2016_3459_MOESM5_ESM.pdf]
