# Supplementary material for: Estimating genomic diversity and population differentiation – an empirical comparison of microsatellite and SNP variation in Arabidopsis halleri
Source: BMC Genomics. 2017 Jan 11;18:69. doi: 10.1186/s12864-016-3459-7 (PMC5225627; doi:10.1186/s12864-016-3459-7)
Supplement: Additional file 6: Figure S2. — Box-whisker plot of expected heterozygosity calculated for each microsatellite marker (SSR-H e) genotyped in Arabidopsis halleri. For cross-species microsatellites (left, red), no significantly different estimates of H e (pairwise Wilcoxon signed-rank test) were observed after Bonferroni correction, as a consequence of the high variance. Without correction for multiple testing, only population Aha11 had a significantly different H e estimates from Aha31 and AhaN1 (indicated with different colouring and letters). For species-specific markers (right, green), no significant differences in H e estimates were observed after Bonferroni correction. Without multiple corrections, only Aha21 was significantly different from AhaN4. (PDF 225 kb) [file 12864_2016_3459_MOESM6_ESM.pdf]

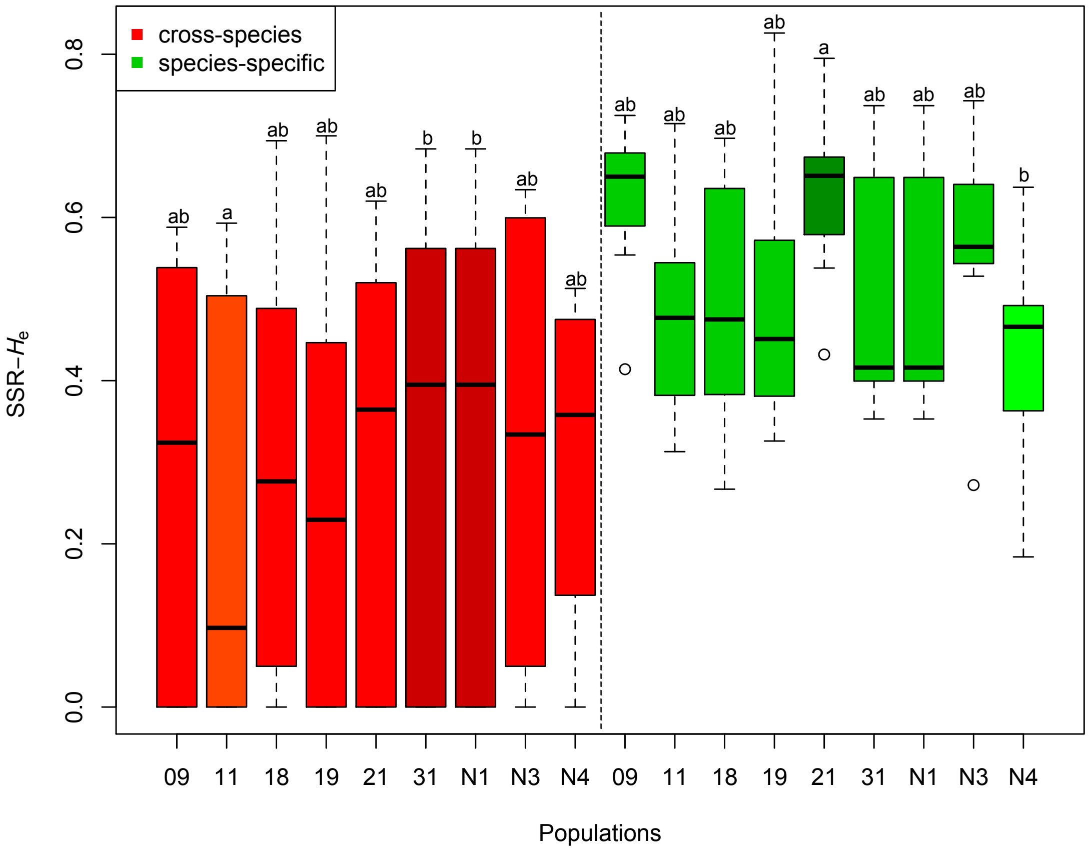

Additional file 6: Figure S2 Box-whisker plot of expected heterozygosity calculated for each microsatellite marker (SSR-H_e) genotyped in *Arabidopsis halleri*. For cross-species microsatellites (left, red), no significantly different estimates of H_e (pairwise Wilcoxon signed-rank test) were observed after Bonferroni correction, as a consequence of the high variance. Without correction for multiple testing, only population Aha11 had a significantly different H_e estimates from Aha31 and AhaN1 (indicated with different colouring and letters). For species-specific markers (right, green), no significant differences in H_e estimates were observed after Bonferroni correction. Without multiple corrections, only Aha21 was significantly different from AhaN4.
