# Supplementary material for: Estimating genomic diversity and population differentiation – an empirical comparison of microsatellite and SNP variation in Arabidopsis halleri
Source: BMC Genomics. 2017 Jan 11;18:69. doi: 10.1186/s12864-016-3459-7 (PMC5225627; doi:10.1186/s12864-016-3459-7)
Supplement: Additional file 7: Figure S3. — Observed (solid red lines) versus expected (dotted black lines) distributions of Tajima’s D for nine populations of Arabidopsis halleri. The expected normal distribution consists of 22,210 values with an average of zero and the same standard deviation as the real dataset. p-values indicate whether there was a significant deviation of the average from zero using a t-test. (PDF 370 kb) [file 12864_2016_3459_MOESM7_ESM.pdf]

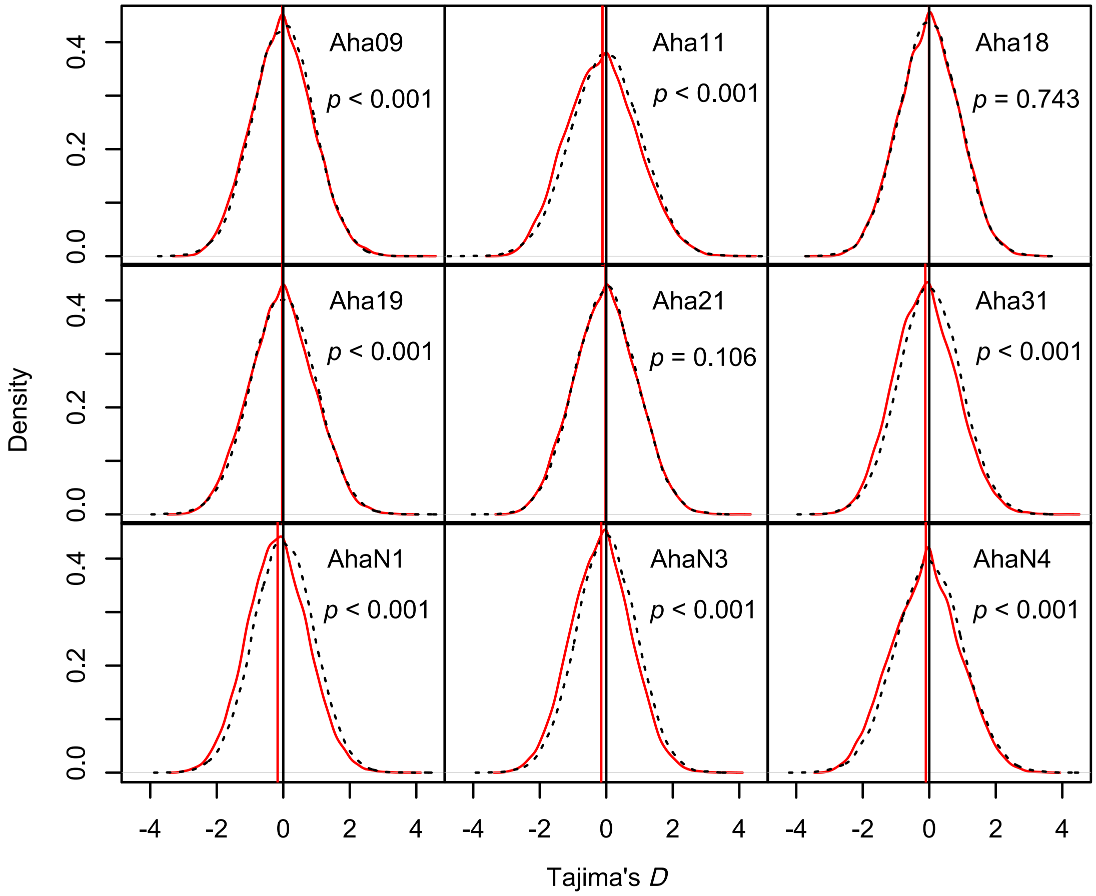


**Additional file 7: Figure S3** Observed (solid red lines) versus expected (dotted black lines) distributions of Tajima's *D* for nine populations of *Arabidopsis halleri*. The expected normal distribution consists of 22,210 values with an average of zero and the same standard deviation as the real dataset. *P*-values indicate whether there was a significant deviation of the average from zero using a *t*-test.
