# Supplementary material for: Estimating genomic diversity and population differentiation – an empirical comparison of microsatellite and SNP variation in Arabidopsis halleri
Source: BMC Genomics. 2017 Jan 11;18:69. doi: 10.1186/s12864-016-3459-7 (PMC5225627; doi:10.1186/s12864-016-3459-7)
Supplement: Additional file 8: Figure S4. — Relationships between expected SNP heterozygosity (SNP-H e) and expected microsatellite heterozygosity (SSR-H e) of Arabidopsis halleri estimated on the basis of 19 microsatellites and 41,294 genetically unlinked genome-wide single nucleotide polymorphisms (SNPs). For this analysis only every 50th SNP of the more than 2 million SNPs was used. The median distance among two neighbouring SNPs is 1600 bp on the A. thaliana reference genome. (PDF 198 kb) [file 12864_2016_3459_MOESM8_ESM.pdf]

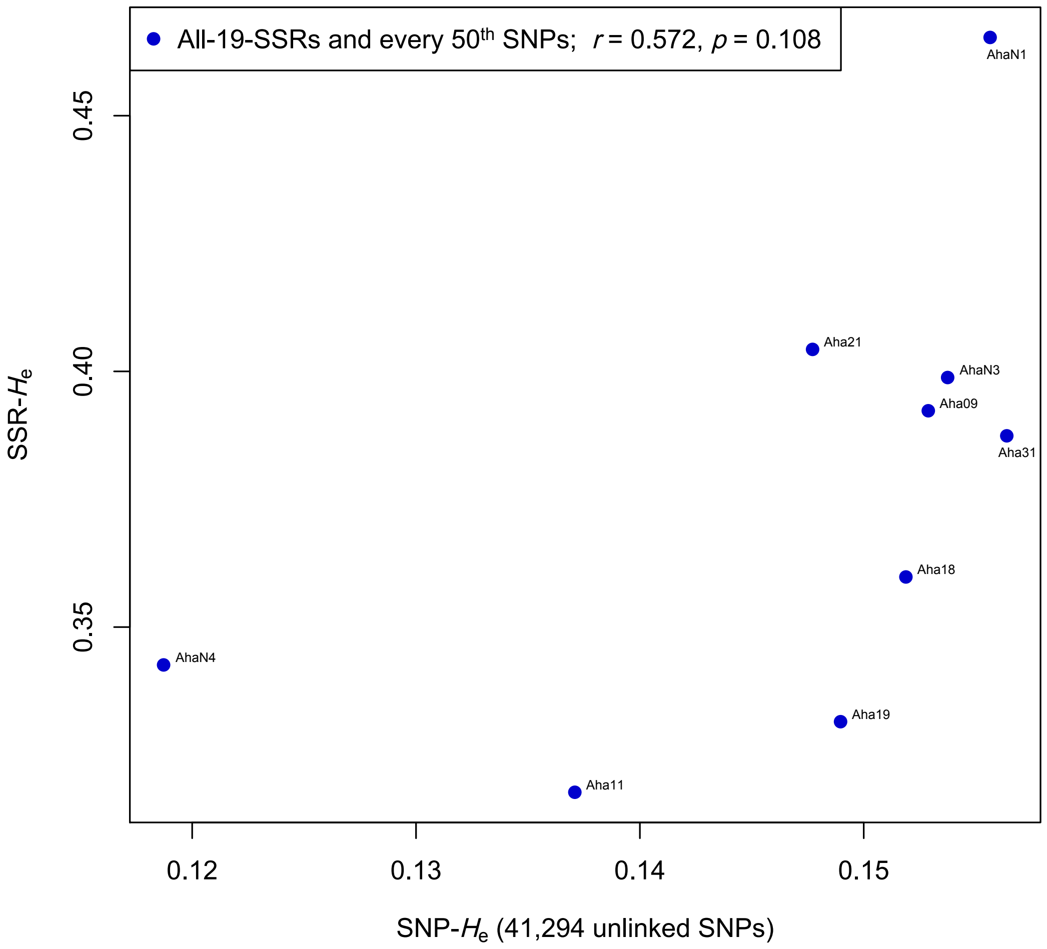

Additional file 8: Figure S4 Relationships between expected SNP heterozygosity (SNP-H_e) and expected microsatellite heterozygosity (SSR-H_e) of *Arabidopsis halleri* estimated on the basis of 19 microsatellites and 41,294 genetically unlinked genome-wide single nucleotide polymorphisms (SNPs). For this analysis only every 50th SNP of the more than 2 million SNPs was used. The median distance among two neighbouring SNPs is 1600 bp on the *A. thaliana* reference genome.
