# Supplementary material for: Estimating genomic diversity and population differentiation – an empirical comparison of microsatellite and SNP variation in Arabidopsis halleri
Source: BMC Genomics. 2017 Jan 11;18:69. doi: 10.1186/s12864-016-3459-7 (PMC5225627; doi:10.1186/s12864-016-3459-7)
Supplement: Additional file 9: Figure S5. — Pearson’s correlation (dashed line) between exome-wide θ Watterson and θ Watterson calculated from introns and intergenic regions in Arabidopsis halleri. (PDF 191 kb) [file 12864_2016_3459_MOESM9_ESM.pdf]

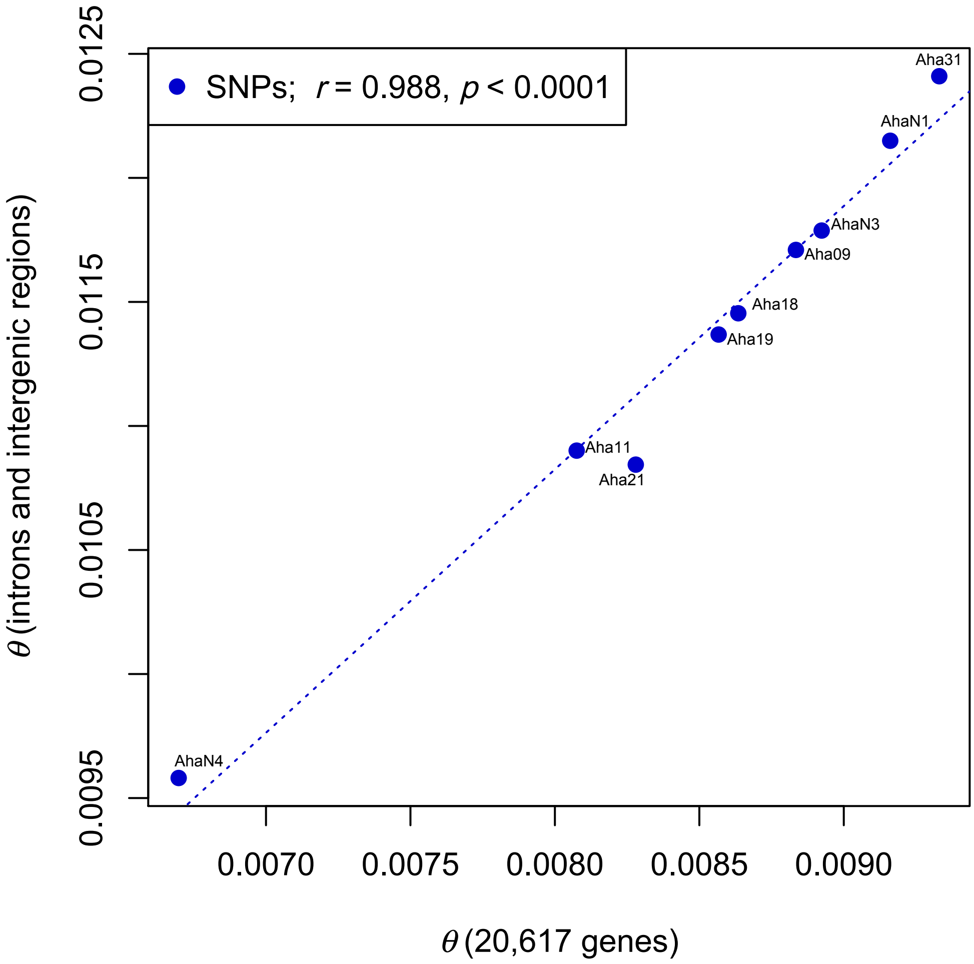

Additional file 9: Figure S5 Pearson's correlation (dashed line) between exome-wide $\theta_{\text{Watterson}}$ and $\theta_{\text{Watterson}}$ calculated from introns and intergenic regions* in *Arabidopsis halleri*.

*Intronic and intergenic $\theta_{\text{Watterson}}$ was calculated by inverting the exon .gtf file to intronic and intergenic regions. We thus obtained $\theta_{\text{Watterson}}$ estimates of 8'143 regions consisting of the intergenic regions upstream of the 5'-ends of genes and their introns. The minimum coverage per site within populations was set to 20x, which mimics the number of individuals. To further correct for potential errors caused by repeated sequences, a maximum coverage of 400x per population was used as upper threshold for SNP identification. In order to be included in the genome-wide estimates of diversity, 50% of SNPs within a region had to reach the above-mentioned thresholds in all nine populations [1]. For all analyses, pool size per population was set to 40 because 20 diploid genomes were represented in each population pool.

REFERENCES

1. Fischer MC, Rellstab C, Tedder A, Zoller S, Gugerli F, Shimizu KK, Holderegger R, Widmer A. Population genomic footprints of selection and associations with climate in natural populations of *Arabidopsis halleri* from the Alps. Mol Ecol. 2013;22:5594-607.
