# Supplementary material for: Estimating genomic diversity and population differentiation – an empirical comparison of microsatellite and SNP variation in Arabidopsis halleri
Source: BMC Genomics. 2017 Jan 11;18:69. doi: 10.1186/s12864-016-3459-7 (PMC5225627; doi:10.1186/s12864-016-3459-7)
Supplement: Additional file 10: Figure S6. — Comparison of pairwise population genetic differentiation (F ST) among nine populations of Arabidopsis halleri based on 12 cross-species microsatellite markers and seven species-specific microsatellite markers. r MT represents the correlation coefficient of the Mantel test and p the significance of the correlation. (PDF 181 kb) [file 12864_2016_3459_MOESM10_ESM.pdf]

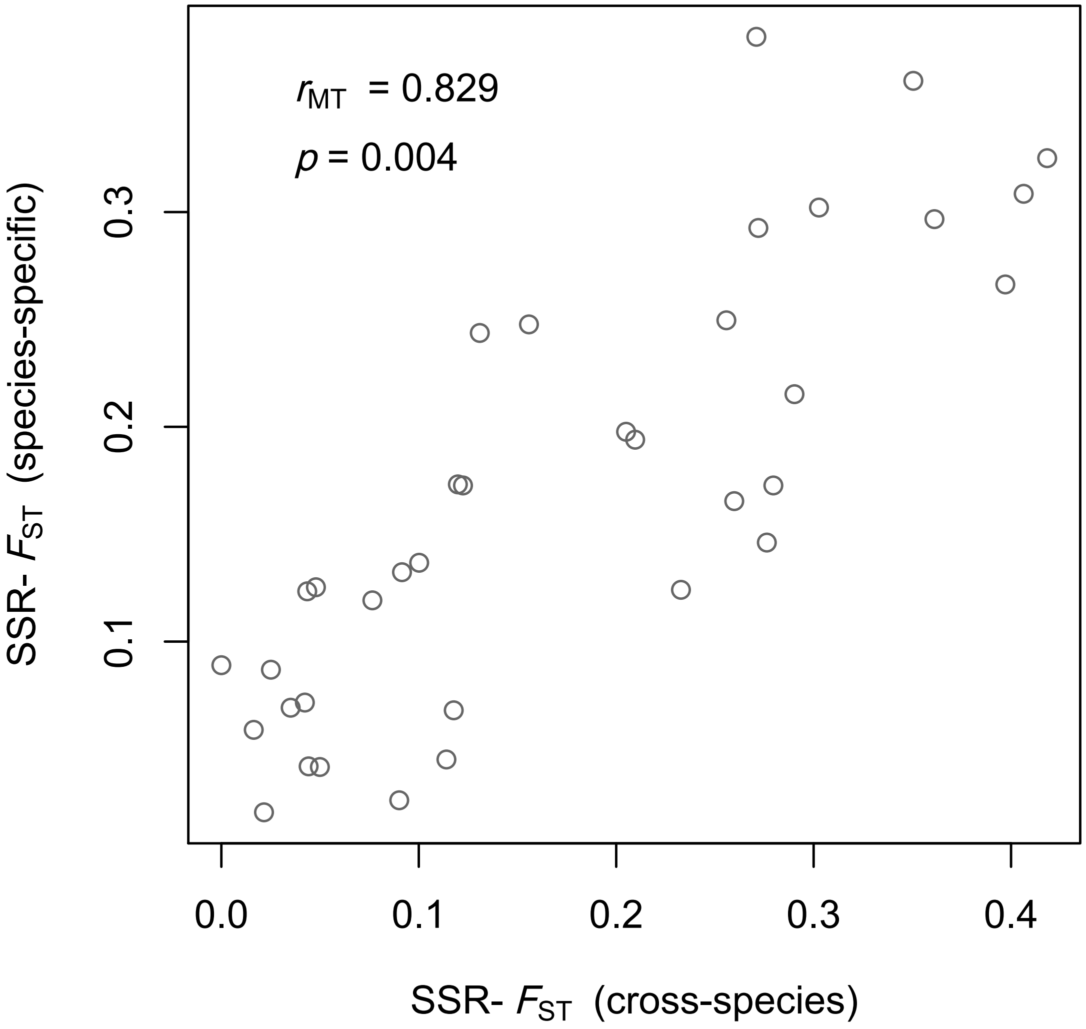

Additional file 10: Figure S6 Comparison of pairwise population genetic differentiation (F_{ST}) among nine populations of *Arabidopsis halleri* based on 12 cross-species microsatellite markers and seven species-specific microsatellite markers. r_{MT} represents the correlation coefficient of the Mantel test and p the significance of the correlation.
